# Anti-Biofilm and Anti-Inflammatory Properties of the Truncated Analogs of the Scorpion Venom-Derived Peptide IsCT against *Pseudomonas aeruginosa*

**DOI:** 10.3390/antibiotics13080775

**Published:** 2024-08-16

**Authors:** Pornpimon Jantaruk, Kittitat Teerapo, Supattra Charoenwutthikun, Sittiruk Roytrakul, Duangkamol Kunthalert

**Affiliations:** 1 Department of Microbiology and Parasitology, Faculty of Medical Science, Naresuan University, Phitsanulok 65000, Thailand; pornpimonj59@nu.ac.th (P.J.); kittitat.nut@gmail.com (K.T.); supattrasptc@gmail.com (S.C.); 2 Functional Proteomics Technology Laboratory, National Center for Genetic Engineering and Biotechnology, National Science and Technology Development Agency, Thailand Science Park, Pathumthani 12120, Thailand; sittiruk@biotec.or.th; 3 Centre of Excellence in Medical Biotechnology, Faculty of Medical Science, Naresuan University, Phitsanulok 65000, Thailand

**Keywords:** anti-biofilm, anti-inflammation, *P. aeruginosa*, pyocyanin, scorpion peptide

## Abstract

*Pseudomonas aeruginosa* is an opportunistic pathogen in humans and a frequent cause of severe nosocomial infections and fatal infections in immunocompromised individuals. Its ability to form biofilms has been the main driving force behind its resistance to almost all conventional antibiotics, thereby limiting treatment efficacy. In an effort to discover novel therapeutic agents to fight *P. aeruginosa*-associated biofilm infections, the truncated analogs of scorpion venom-derived peptide IsCT were synthesized and their anti-biofilm properties were examined. Among the investigated peptides, the IsCT-Δ6-8 peptide evidently showed the most potential anti-*P. aeruginosa* biofilm activity and the effect was not due to bacterial growth inhibition. The IsCT-Δ6-8 peptide also exhibited inhibitory activity against the production of pyocyanin, an important virulence factor of *P. aeruginosa*. Furthermore, the IsCT-Δ6-8 peptide significantly suppressed the production of inflammatory mediators nitric oxide and interleukin-6 in *P. aeruginosa* LPS-induced macrophages. Due to its low cytotoxicity to mammalian cells, the IsCT-Δ6-8 peptide emerges as a promising candidate with significant anti-biofilm and anti-inflammatory properties. These findings highlight its potential application in treating *P. aeruginosa*-related biofilm infections.

## 1. Introduction

*Pseudomonas aeruginosa* is a Gram-negative human opportunistic pathogen that has become a common cause of nosocomial infections, especially in immunocompromised patients [1,2]. *P. aeruginosa* exhibits high intrinsic resistance to common antimicrobial agents and this has substantially limited the efficacy of currently used antibiotics [3,4,5]. The treatment of *P. aeruginosa* infections is further complicated by the ability of this microorganism to form biofilms which render it tolerant to antibiotics, impede the host defense system, and confer the ability for colonization and long-term persistence [6,7]. The rise in multidrug resistance of *P. aeruginosa* has been closely associated with the establishment of chronic persistent infections [8,9,10]. This perspective therefore highlights an urgent need to develop alternative therapeutics to fight against biofilm-associated *P. aeruginosa* infections. The World Health Organization has listed *P. aeruginosa* as a critical priority pathogen for new antibiotic development [11].

Antimicrobial peptides (AMPs) are a class of short peptides produced by a wide variety of living organisms, serving as crucial components of the innate immune system [12]. AMPs have received considerable attention as a promising class of antibiotics because of their potency, rapid onset of action [13], and broad-spectrum biological properties including anti-microbial, anti-tumor and anti-inflammatory activities [12,14,15,16]. Low potential to cause bacterial resistance is also an interesting feature of AMPs [17,18,19]. Significantly, AMPs can be modified by adding, deleting, or replacing amino acid residues in order to obtain the desired biological features [20,21]. These distinctive properties of AMPs have therefore made them highly attractive for therapeutic purposes.

Scorpion venom is a heterogenous mixture containing a diverse array of bioactive molecules, including AMPs. IsCT is an α-helical AMP comprising only 13 amino acid residues (ILGKIWEGIKSLF-NH_2_) which was originally isolated from the venom of the scorpion *Opisthacanthus madagascariensis* [22,23]. IsCT exhibits potent antimicrobial properties against Gram-positive and Gram-negative bacteria, but also shows cytolytic activity against mammalian cells [23]. Due to its non-cell-selective nature, efforts have been made to modify this peptide to improve its selectivity for bacterial cells and minimize toxicity. Several IsCT analogs have been designed and synthesized. For instance, five IsCT analogs, designated [A]^6^-IsCT, [L]^6^-IsCT, [K]^7^-IsCT, [L]^6^[K]^7^-IsCT, and [K]^7^[P]^8^[K]^11^-IsCT, were designed and synthesized [24]. It was found that the [K]^7^[P]^8^[K]^11^-IsCT peptide, which had a bend in its middle region, exhibited the highest antibacterial activity without hemolytic activity [24]. In another study, I9K-IsCT, E7K-IsCT and E7K, I9K-IsCT analogs were designed to investigate the impact of introducing positive charges to polar faces, as well as to both polar and non-polar faces simultaneously, on antibacterial activity [25]. These amino acid substitutions, particularly the addition of positive charges to both polar and non-polar faces at the same time, resulted in a significant improvement in antibacterial activity with very low toxicity, thereby enhancing the therapeutic potential of IsCT [25]. In a recent study, five IsCT-based analogs with amino acid modifications at positions 1, 3, 5, or 8 ([W]^1^-IsCT1-NH_2_, [L]^3^-IsCT1-NH_2_, [K]^3^-IsCT1-NH_2_, [F]^5^-IsCT1-NH_2_, [P]^8^-IsCT1-NH_2_,) and one analog with three simultaneous substitutions at positions 1, 5, and 8 ([A]^1^[F]^5^[K]^8^-IsCT1-NH_2_) were designed [26]. The authors observed that among the studied peptides, [A]^1^[F]^5^[K]^8^-IsCT1-NH_2_, which carried three simultaneous modifications, displayed an increase in antimicrobial activity with a reduction in hemolytic activity [26].

The previously described IsCT-based analogs show promising biological properties, suggesting their potential in the development of novel peptide therapeutics. This study further explores the therapeutic potential of IsCT-based analogs by examining their anti-*P. aeruginosa* biofilm and anti-inflammatory properties. Five new IsCT-based peptides were developed based on the previous findings, which revealed that a positively charged lysine (K) substitution at position 7 in the K7-IsCT analog improved antibacterial activity, particularly against *P. aeruginosa* [24,25]. However, this substitution did not reduce toxicity to mammalian cells, as approximately 50% of the hemolytic activity of the native IsCT remained [24,27]. Toxicity and the ability to lyse mammalian cells are major barriers to the use of AMPs as therapeutics. Accordingly, we hypothesize that the removal of the positively charged lysine at position 7 and its neighboring amino acids would reduce this toxicity. Therefore, truncated analogs of IsCT were synthesized, and their effects on toxicity as well as anti-*P. aeruginosa* biofilm and anti-inflammatory properties were investigated.

## 2. Results

### 2.1. Hemolytic Activity of the Truncated IsCT Analogs

To evaluate the toxicity of the truncated analogs of IsCT, a hemolytic assay was performed using sheep erythrocytes. As presented in Table 1, the incubation of red blood cells with the truncated IsCT analogs resulted in very low levels of hemolysis. This minimal hemolytic activity was observed for all the studied peptides, even at concentrations as high as 150 μM.

### 2.2. Anti-Biofilm Activities of the Truncated IsCT Analogs against P. aeruginosa

**Figure 1:**
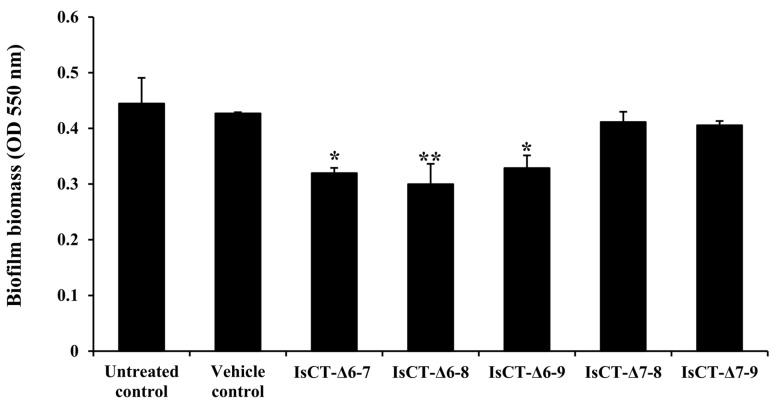
The effects of the truncated IsCT analogs against *P. aeruginosa* biofilms. *P. aeruginosa* culture cells were incubated in the absence or presence of the truncated IsCT analogs (final concentration, 150 μM) at 37 °C for 24 h. Biofilm biomass was determined by crystal violet staining and the OD was measured at 550 nm. Values are expressed as the mean ± SEM of two independent experiments performed in duplicate. *, *p* < 0.05; **, *p* < 0.01 compared with the untreated control.

Anti-biofilm activities of the truncated IsCT analogs against *P. aeruginosa* were investigated using a crystal violet assay. The results in Figure 1 illustrate that biofilm biomass of *P. aeruginosa* was decreased in the presence of the truncated IsCT analogs compared with the untreated control. Despite the varying degrees of reduction, significant decreases in *P. aeruginosa* biofilm biomass were observed with the IsCT-Δ6-8 peptide (33.12% decrease), followed by IsCT-Δ6-7 (28.27%) and IsCT-Δ6-9 peptides (26.45%). The anti-biofilm activities were less pronounced with IsCT-Δ7-8 and IsCT-Δ7-9 peptides. No significant differences were observed between the vehicle and untreated controls. Since the IsCT-Δ6-8 peptide demonstrated the most anti-biofilm activity while exhibiting very low hemolytic effect, this peptide was selected for further testing.

### 2.3. Effects of the IsCT-Δ6-8 Peptide on Growth of P. aeruginosa

**Figure 2:**
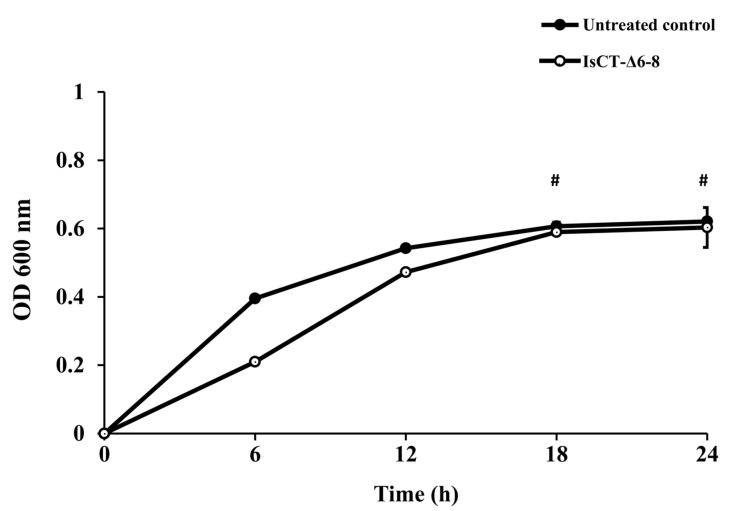
The effects of the IsCT-Δ6-8 peptide on the growth of *P. aeruginosa*. The culture of *P. aeruginosa* was treated with the IsCT-Δ6-8 peptide at a final concentration of 150 μM. The bacterial culture alone served as an untreated control. The cultures were incubated at 37 °C and bacterial growth was assessed by measuring OD600 at the indicated time points. Values are expressed as the mean ± SEM of two independent experiments performed in duplicate. #, not statistically different to the untreated control (*p* > 0.5).

To determine whether the observed inhibitory activity against *P. aeruginosa* biofilms by IsCT-Δ6-8 peptide was related to its growth-inhibitory effect, a culture-based growth assay of *P. aeruginosa* in the presence of the IsCT-Δ6-8 peptide was carried out. As presented in Figure 2, a slow growth rate of *P. aeruginosa* in the presence of the IsCT-Δ6-8 peptide was observed at the early time points (6 h and 12 h) as compared to the untreated control culture. However, the *P. aeruginosa* culture continued to grow after prolonged exposure (18 h and 24 h) to the test peptide; the growth curve appeared to be comparable to that of the untreated control culture (*p* > 0.05). These results suggested that the decrease in *P. aeruginosa* biofilms by the IsCT-Δ6-8 peptide did not result from its growth-inhibitory effect.

### 2.4. Effects of the IsCT-Δ6-8 Peptide on Pyocyanin Production of P. aeruginosa

**Figure 3:**
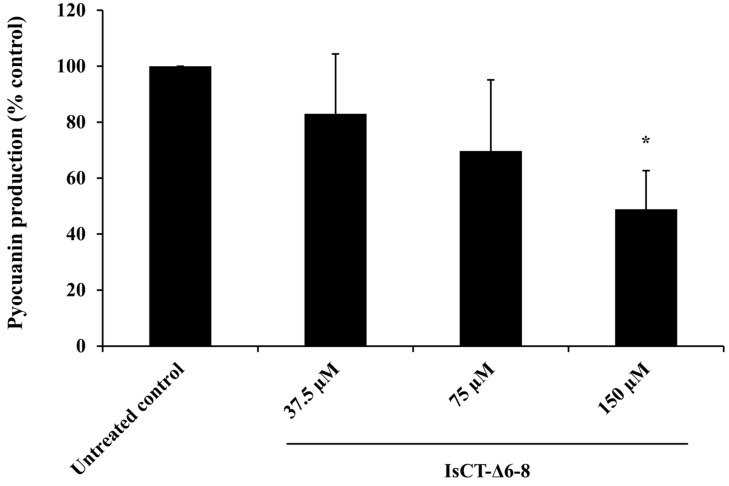
Effects of the IsCT-Δ6-8 peptide on pyocyanin production in *P. aeruginosa*. A culture of *P. aeruginosa* was treated with varying concentrations of the IsCT-Δ6-8 peptide (37.5, 75 and 150 μM) for 24 h, and pyocyanin levels were quantified using the chloroform extraction method. Values are expressed as mean ± SEM of two independent experiments performed in duplicate. *, *p* < 0.05 compared with the untreated control.

Pyocyanin is a major virulence factor produced by *P. aeruginosa* during host infection and its production is closely related to the formation process of *P. aeruginosa* biofilms [28]. The effect of the IsCT-Δ6-8 peptide on *P. aeruginosa* pyocyanin production was additionally examined. As presented in Figure 3, it was found that IsCT-Δ6-8 peptide, depending on dosage, decreased pyocyanin production by *P. aeruginosa*, with inhibition ranging from 17.03% to 51.09%.

### 2.5. Effects of the IsCT-Δ6-8 Peptide on the Viability of RAW 264.7 Macrophages

**Figure 4:**
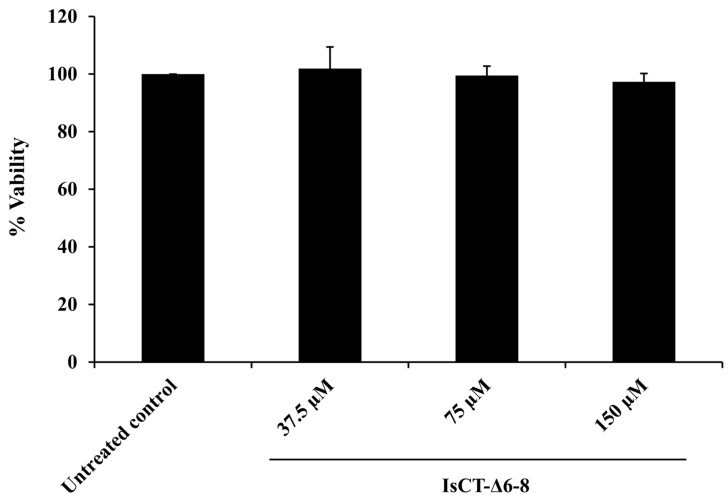
Effects of the IsCT-Δ6-8 peptide on the viability of RAW 264.7 macrophages. RAW 264.7 macrophage cells were treated with various concentrations of the IsCT-Δ6-8 peptide (37.5, 75 and 150 μM) for 48 h. Cell viability was assessed by the MTT assay.

Prior to investigating the anti-inflammatory activity of the IsCT-Δ6-8 peptide, its cytotoxic effect on RAW 264.7 macrophages was determined using an MTT assay. It was found that the viability of RAW 264.7 macrophages exposed to the IsCT-Δ6-8 peptide at 37.5–150 μM was not significantly changed as compared with the untreated control (Figure 4). These results indicated that the IsCT-Δ6-8 peptide at the studied concentrations did not induce significant cellular cytotoxicity, so these concentrations were employed in anti-inflammatory experiments.

### 2.6. Effects of the IsCT-Δ6-8 Peptide on Nitric Oxide (NO) Production in P. aeruginosa LPS-Stimulated RAW 264.7 Macrophages

**Figure 5:**
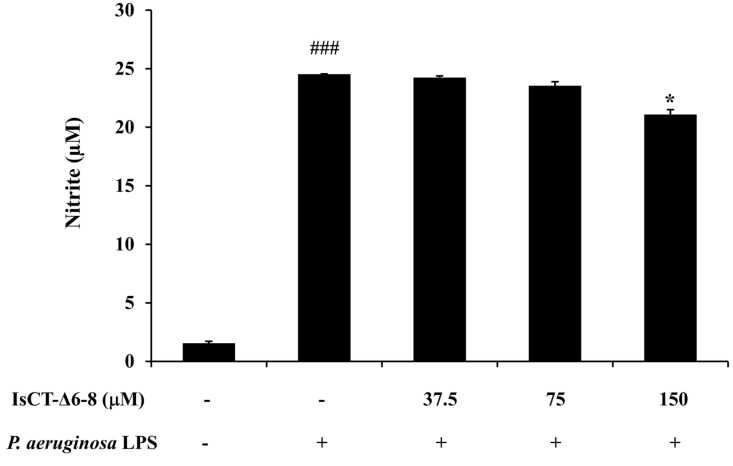
Effects of the IsCT-Δ6-8 peptide on NO production in *P. aeruginosa* LPS-stimulated RAW 264.7 macrophages. RAW 264.7 macrophages were incubated with *P. aeruginosa* LPS in the presence or absence of the IsCT-Δ6-8 peptide (37.5–150 μM) for 48 h at 37 °C under 5% CO_2_. Nitrite levels in the culture supernatant were determined by Griess reagents. ###, *p* < 0.001 compared with unstimulated RAW 264.7 macrophages; *, *p* < 0.05 compared with the *P. aeruginosa* LPS-stimulated RAW 264.7 macrophages.

To investigate the anti-inflammatory activity of the IsCT-Δ6-8 peptide, its effect on the production of the important inflammatory mediator NO in *P. aeruginosa* LPS-stimulated RAW 264.7 macrophages was determined. The results from the Griess assay in Figure 5 showed that nitrite concentration markedly increased in RAW 264.7 macrophages stimulated with *P. aeruginosa* LPS compared with the untreated control. However, this effect was reduced by treatment with the IsCT-Δ6-8 peptide, in particular at the peptide concentration of 150 μM.

### 2.7. Effects of the IsCT-Δ6-8 Peptide on IL-6 Production in P. aeruginosa LPS-Stimulated RAW 264.7 Cells

**Figure 6:**
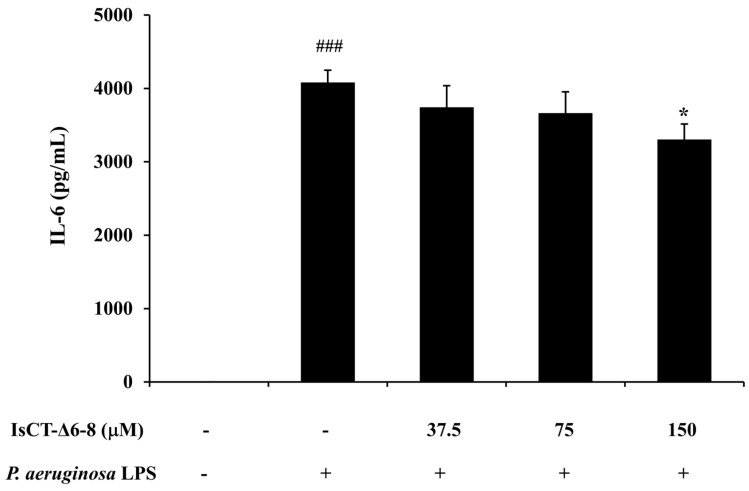
Effects of the IsCT-Δ6-8 peptide on IL-6 production in *P. aeruginosa* LPS-stimulated RAW 264.7 macrophages. RAW 264.7 macrophages were incubated with *P. aeruginosa* LPS in the presence or absence of IsCT-Δ6-8 peptide (37.5–150 μM) for 48 h at 37 °C under 5% CO_2_. The level of IL-6 in culture supernatant was measured by sandwich ELISA. ###, *p* < 0.001 compared with the unstimulated RAW 264.7 macrophages; *, *p* < 0.05 compared with the *P. aeruginosa* LPS-stimulated RAW 264.7 macrophages.

To further assess the anti-inflammatory potential of the IsCT-Δ6-8 peptide, its effect on IL-6 production in *P. aeruginosa* LPS-induced RAW 264.7 macrophages was also determined, and this was performed by sandwich ELISA. The results in Figure 6 demonstrated a significant elevation of IL-6 level in *P. aeruginosa* LPS-induced RAW 264.7 macrophages as compared with the untreated control, which was reduced by treatment of the IsCT-Δ6-8 peptide, especially at the concentration of 150 μM.

## 3. Discussion

*P. aeruginosa* is known to play an important role in life-threatening and hospital-acquired infections. Its ability to form biofilms makes it difficult to eradicate with conventional antibiotic therapy. This study aimed to discover novel molecules with anti-biofilm and anti-inflammatory activities that are effective against *P. aeruginosa* while ensuring minimal toxicity. Previous research suggested that the positively charged lysine at position 7 in K7-IsCT peptide (ILGKIWKGIKSLF) was responsible for the lysis of mammalian erythrocytes [24,27]. We therefore synthesized five truncated analogs of IsCT, in which the positively charged lysine at position 7 and adjacent amino acids were removed. We then assessed their hemolytic activity, as hemolysis against normal red blood cells is a common initial toxicity assessment for AMPs [29]. Our findings showed that the truncated IsCT analogs IsCT-Δ6-7, IsCT-Δ6-8, IsCT-Δ6-9, IsCT-Δ7-8, and IsCT-Δ7-9 exhibited decreased hemolytic activity, even at peptide concentrations as high as 150 μM. This suggested that these developed peptides have low cytotoxicity towards mammalian cells. The physicochemical analysis of the truncated IsCT analogs revealed that the removal of the positively charged lysine at position 7 and its neighboring amino acids decreased the net charge and hydrophobic moment while increasing hydrophobicity (Table 2) compared to the K7-IsCT template (which had a net charge of +3, a hydrophobic moment of 0.803, and hydrophobicity of 0.756). It is well established that the net charge, hydrophobicity, and hydrophobic moment of cationic antimicrobial peptides are directly associated with hemolytic activity [30,31,32]. Consequently, the decreased net charge and hydrophobic moment of the truncated IsCT analogs would likely contribute to the dramatically reduced hemolytic activity observed in this study.

We next determined the anti-biofilm potentials of the truncated IsCT analogs against biofilms formation of *P. aeruginosa*. Our findings demonstrated that among the studied peptides, the IsCT-Δ6-8 peptide displayed the most promising biofilm-inhibitory activity. Our results also showed that the biofilm-inhibitory activity of the IsCT-Δ6-8 peptide was not due to its growth-inhibitory effect. Antibacterials with growth-inhibitory activity may accelerate the evolutionary pressure and ultimately lead to troublesome antibiotic resistance [33]. Several studies have demonstrated that various AMPs can reduce *P. aeruginosa* biofilm biomass, with inhibition rates ranging from 20% to 70% [34,35,36,37]. For instance, peptide P5 inhibited the biofilm formation of *P. aeruginosa* by approximately 20% at 0.5 × MIC [38], while an AMP β-defensin 2 inhibited the biofilm production of *P. aeruginosa* by 40% at a 4 μM concentration [37]. Peptides LL-31 and LL7-37, derived from the human AMP LL-37, were able to inhibit *P. aeruginosa* biofilm formation by 70% at 10 μM [36]. Additionally, a study on the scorpion venom peptide derivative BmKn–22 found that the BmKn–22 peptide effectively inhibited *P. aeruginosa* biofilm formation by approximately 49% at 800 μM [39]. Although the 33% biofilm inhibition observed with the IsCT-Δ6-8 peptide in this study is lower compared to some other AMPs reported previously, it is important to note that this peptide still exhibited moderate inhibitory activity. This indicates some potential for reducing biofilm formation. In addition to biofilm-inhibitory activity, the IsCT-Δ6-8 peptide also inhibited pyocyanin production by *P. aeruginosa*. Pyocyanin is recognized as one of the most potent virulence factors contributing to the pathogenesis of *P. aeruginosa*. This blue redox-active secondary metabolite can produce reactive oxygen species and interferes directly with multiple cellular functions. Significantly, pyocyanin plays an essential role in promoting the development of *P. aeruginosa* biofilms [28], which occurs via the release of extracellular DNA through hydrogen peroxide-mediated cell lysis [40]. A significant positive correlation was observed between pyocyanin production and the biofilm formation of *P. aeruginosa* [41]. Previous studies have also reported that large quantities of pyocyanin were recovered from the sputum of patients with cystic fibrosis who were *P. aeruginosa*-infected, and this correlated with disease severity [42]. Biofilm formation and pyocyanin production have been considered as potential targets for new therapeutic development against *P. aeruginosa* infection [41,43] and the IsCT-Δ6-8 peptide represents a promising candidate in this regard.

During infection, biofilm-forming *P. aeruginosa* triggers the host immune response and induces high production rates of inflammatory mediators [44,45]. It has been reported that biofilm matrix exopolysaccharides (EPS) and eDNA from *P. aeruginosa* can induce the production of NO, IL-6, and tumor necrosis factor-α in RAW 264.7 macrophage cells [46]. Recent investigations have also revealed that LPS obtained from biofilm-forming *P. aeruginosa* PAO1 induced significantly greater hyperinflammatory responses in human and macrophage cell lines than LPS in its planktonic form [47]. The excessive or uncontrolled production of inflammatory mediators can result in severe damage to airways and eventually decrease lung function [48]. Accordingly, the anti-inflammatory properties of the IsCT-Δ6-8 peptide were also investigated in this study. This was performed using the *P. aeruginosa* LPS-induced RAW 264.7 macrophage cell line as a model of inflammation, and the representative inflammatory mediators NO and IL-6 were examined. NO is a central mediator of pathogenesis in infection [49], while IL-6 is a pro-inflammatory cytokine that results in the occurrence of rapid and transient tissue damage in response to infection [50]. Here, we observed reduced levels of both NO and IL-6 in *P. aeruginosa* LPS-induced RAW 264.7 macrophages upon treatment with the IsCT-Δ6-8 peptide, suggesting its anti-inflammatory properties. Taken together, our results clearly demonstrate that the IsCT-Δ6-8 peptide, a truncated form of a scorpion-derived antimicrobial peptide IsCT, possesses dual action in terms of biofilm inhibition and anti-inflammatory activity against *P. aeruginosa*, while simultaneously displaying low cytotoxicity. Our findings provide an important step in combating *P. aeruginosa*-related biofilm infections. However, the mechanisms by which the IsCT-Δ6-8 peptide inhibits biofilms and inflammation induced by *P. aeruginosa* require further investigation.

## 4. Materials and Methods

### 4.1. Truncated IsCT Analogs and Their Physicochemical Properties

Truncated IsCT analogs, with a purity > 95%, were obtained from ChinaPeptides Co., Ltd. (Shanghai, China) or GenScript (Piscataway, NJ, USA). The amino acid sequences and the physiochemical properties of the studied peptides are shown in Table 2. The molecular weights and net charges were computed by the APD3: Antimicrobial Peptide Calculator and Predictor [51], while the isoelectric point (pI) was from INNOVAGEN Peptide property calculator [52]. Hydrophobicity (H) and hydrophobic moment (M) were estimated by the HeliQuest [53]. All studied peptides were solubilized in their vehicle, dimethyl sulfoxide (DMSO; >99.5%, Sigma, Lyon France), and further diluted in the appropriate culture medium to the required working concentrations.

### 4.2. Hemolytic Assay

A hemolytic assay was carried out according to the previously described method [24] using commercially sourced sheep red blood cells (Professional Nanomed Company Limited, Bangkok, Thailand). One hundred microliters of a 2% sheep red blood cell suspension prepared in phosphate-buffered saline (PBS) pH 7.4. was incubated for 1 h at 37 °C with 100 μL of the studied peptides (final concentration of 150 µM). After centrifugation at 1000× *g* for 5 min, 100 μL of supernatant was transferred to new 96-well plates, and the release of hemoglobin was monitored, measuring an optical density (OD) at 405 nm using a BioTek Synergy HT microplate reader. Red blood cells treated with PBS pH 7.4 were used as a negative control, while those treated with 1% Triton X-100 were used as a positive control. The percent hemolysis was calculated as follows:

(1) $$\%\;\mathrm{Hemolysis}=\left.\left(\frac{OD405\;nm\;of\;the\;test\;peptide-OD405\;nm\;of\;negative\;control}{OD405\;nm\;of\;positive\;control-OD405\;nm\;of\;negative\;control}\right.\right)\;\times \;100$$

### 4.3. Bacterial Strain and Culture Condition

This investigation used the bacterial strain *P. aeruginosa* PAO1, which was purchased from Spanish Type Culture Collection (Valencia, Spain). This bacterial strain was cultivated on Luria-Bertani (LB) agar (BD Difco^TM^, Sparks, Maryland, USA) and incubated at 37 °C for 24 h under aerobic conditions. For subsequent experiments, a single colony on LB agar was taken and a bacterial suspension was prepared in an appropriate culture medium.

### 4.4. Biofilm Susceptibility Assay

The effect of the truncated IsCT analogs on biofilms of *P. aeruginosa* was assessed using a method that was described previously [39,54]. Briefly, 100 μL of the test peptides (final concentration, 150 μM) was added to each well of a 96-well microtiter plate (Nunc^TM^, Roskilde, Denmark). Then, an aliquot of *P. aeruginosa* PAO1 culture was added to each well to obtain a final concentration of 10^6^ CFU/mL. The bacterial culture alone was included as an untreated control. Following a 24-h incubation at 37 °C, unattached planktonic cells were discarded, and the wells were carefully washed twice with PBS with a pH of 7.4. Thereafter, biofilm biomasses were quantified by 0.1% crystal violet staining. After excess stains were discarded, the wells were washed with PBS pH 7.4 and left to air dry for 2 h at 37 °C. The biofilm biomass was subsequently dissolved by the addition of 30% acetic acid, and the plates were spectrophotometrically read at 550 nm using a BioTek Synergy HT microplate reader.

### 4.5. Growth Curve Assay

The effect of the IsCT-Δ6-8 peptide on the growth of *P. aeruginosa* was assessed as previously described [54]. A culture of *P. aeruginosa* PAO1 was grown at a concentration of 10^6^ CFU/ mL in the presence or absence of the IsCT-Δ6-8 peptide (final concentration, 150 µM) at 37 °C, with agitation at 150 rpm. The OD600 nm was then recorded at 0, 6, 12, 18, and 24 h.

### 4.6. Pyocyanin Assay

The effect of IsCT-Δ6-8 peptide on the production of pyocyanin pigments produced by *P. aeruginosa* PAO1 was assessed using the protocol described previously [55]. In brief, 750 μL of *P. aeruginosa* culture (10^8^ CFU/mL, final concentration) and 250 μL of the test peptide prepared in LB broth (final concentrations ranged from 37.5 μM to 150 μM) were incubated at 37 °C for 24 h with agitation (150 rpm). A *P. aeruginosa* culture without the IsCT-Δ6-8 peptide served as an untreated control. Thereafter, supernatants were collected by centrifugation at 3900× *g* for 10 min, and pyocyanin pigment was extracted twice using chloroform/0.2 N HCl. Centrifugation was performed at 3900× *g* for 10 min before the pink-colored solution was collected, and the absorbance was measured at 380 nm.

### 4.7. Cell Cultures

The murine macrophage cell line RAW264.7 (TIB-71) was obtained from American Type Culture Collection (ATCC; Manassas, VA, USA). RAW 264.7 cells were cultured in DMEM (HyClone^TM^, Logan, UT, USA) supplemented with 10% (*v*/*v*) heat-inactivated fetal bovine serum (Gibco, Fisher Scientific, Göteborg, Sweden), 2 mM L-glutamine (PAA Laboratories GmbH, Pasching, Austria), 10 mM 4-(2-hydroxyethyl)-1-piperazineethanesulfonic acid (HyClone^TM^, Logan, UT, USA), 100 U/mL penicillin, and 100 µg/mL streptomycin (PAA). RAW 264.7 cells were grown in a 5% CO_2_ at 37 °C.

### 4.8. Cytotoxicity Assay

The cytotoxic effect of the IsCT-Δ6-8 peptide was assessed using the tetrazolium-based 3-(4,5-dimethylthiazol-2-yl)-2,5-diphenyltetrazolium bromide (MTT) assay [56]. RAW 264.7 cells at 3.5 × 10^5^ cells were seeded in a 96-well microtiter plate and incubated at 37 °C for 1 h under 5% CO_2_. Then, the cells were treated with or without the IsCT-Δ6-8 peptide at concentrations ranging from 37.5 μM to 150 μM for 48 h at 37 °C under 5% CO_2_. After incubation, 20 µL of MTT (5 mg/mL) was added in each well, and the plate was incubated for another 3 h. The supernatant was then discarded, and 100 µL of DMSO was added to solubilize formazan crystals. The plate was subsequently read at OD540 nm using a BioTek Synergy HT microplate reader. The percent cell viability was calculated using the following equation:

(2) $$\%\;\mathrm{Cell}\;\mathrm{viability}=\left.\left(\frac{OD\;of\;treated\;cells}{OD\;of\;untreated\;cells}\right.\right)\times 100$$

### 4.9. Measurements of Nitric Oxide (NO) and Interleukin (IL)-6

RAW 264.7 cells (3.5 × 10^5^) were stimulated with *P. aeruginosa* lipopolysaccharide (100 ng/mL; Sigma, St. Louis, MO, USA) in the presence or absence of the IsCT-Δ6-8 peptide (37.5–150 μM, final concentration). After incubation at 37 °C under 5% CO_2_ for 48 h, supernatants were collected. The levels of nitrite (a stable breakdown of NO) were then determined using the Griess reagent system (Promega, Madison, Wisconsin, USA). The IL-6 levels in culture supernatant were quantified with the BioLegend ELISA MAXTM Deluxe Set according to the manufacturer’s protocols.

### 4.10. Statistical Analysis

Data are shown as mean ± standard error of mean (SEM) of independent experiments. Statistical analysis was performed by one-way ANOVA or two-tailed Student’s *t*-test and *p* < 0.05 was defined as significant difference.

## 5. Conclusions

This study is the first to demonstrate that the IsCT-Δ6-8 peptide, a truncated analog of the scorpion-derived antimicrobial peptide IsCT, represents a new class of molecule with significant anti-biofilm and anti-inflammatory activities against *P. aeruginosa*. Given that this peptide showed very low cytotoxicity, we propose that the IsCT-Δ6-8 peptide could be a promising candidate for the treatment of *P. aeruginosa* infections associated with biofilms. Our findings also provide a foundation for developing more effective inhibitors. Future research could focus on structural modifications, the optimization of peptide concentration, and exploring delivery methods to enhance the biological properties of IsCT-Δ6-8.

## Figures and Tables

**Table 1:** Hemolytic activity of the truncated IsCT analogs.

Treatment	% Hemolysis ^a^
Untreated control	0.00 ± 0.00
1% Triton X-100	100.00 ± 0.00
IsCT-Δ6-7, 150 μM	0.83 ± 0.81
IsCT-Δ6-8, 150 μM	1.91 ± 0.81
IsCT-Δ6-9, 150 μM	0.47 ± 0.30
IsCT-Δ7-8, 150 μM	2.13 ± 1.00
IsCT-Δ7-9, 150 μM	0.44 ± 0.35

^a^ The results represent mean ± SEM of independent experiments.

**Table 2:** Amino acid sequences and the physicochemical properties of the truncated analogs of IsCT.

Peptides	Amino Acid Sequence	No. of Amino Acid	Molecular Weight (Da)	Net Charge (z)	pI	Hydrophobicity (H)	Hydrophobic Moment (mH)
IsCT-Δ6-7	ILGKIGIKSLF	11	1188.51	+2	10.73	0.779	0.171
IsCT-Δ6-8	ILGKIIKSLF	10	1131.46	+2	10.73	0.857	0.187
IsCT-Δ6-9	ILGKIKSLF	9	1018.30	+2	10.73	0.752	0.491
IsCT-Δ7-8	ILGKIWIKSLF	11	1317.67	+2	10.73	0.984	0.376
IsCT-Δ7-9	ILGKIWKSLF	10	1204.51	+2	10.73	0.902	0.214

## Data Availability

The data that support the findings of this study are included in the Supplementary Materials.

## Supplementary Materials

The following supporting information can be downloaded at: https://www.mdpi.com/article/10.3390/antibiotics13080775/s1, File S1: Raw Data.
